# Nanotechnology-Based Detection and Targeted Therapy in Cancer: Nano-Bio Paradigms and Applications

**DOI:** 10.3390/cancers3032888

**Published:** 2011-07-15

**Authors:** Shaker A. Mousa, Dhruba J. Bharali

**Affiliations:** 1. The Pharmaceutical Research Institute at Albany College of Pharmacy and Health Sciences, 1 Discovery Drive, Rensselaer, NY 12144, USA; E-Mail: dhruba.bharali@acphs.edu; 2. College of Medicine, King Saud University, Riyadh, Saudi Arabia

**Keywords:** nanomedicine, nanoparticles, dendrimer, PLGA, quantum dots, oncology, site-directed delivery, early detection, therapy

## Abstract

The application of nanotechnology to biomedicine, particularly in cancer diagnosis and treatment, promises to have a profound impact on healthcare. The exploitation of the unique properties of nano-sized particles for cancer therapeutics is most popularly known as nanomedicine. The goals of this review are to discuss the current state of nanomedicine in the field of cancer detection and the subsequent application of nanotechnology to treatment. Current cancer detection methods rely on the patient contacting their provider when they feel ill, or relying on non-specific screening methods, which unfortunately often result in cancers being detected only after it is too late for effective treatment. Cancer treatment paradigms mainly rely on whole body treatment with chemotherapy agents, exposing the patient to medications that non-specifically kill rapidly dividing cells, leading to debilitating side effects. In addition, the use of toxic organic solvents/excipients can hamper the further effectiveness of the anticancer drug. Nanomedicine has the potential to increase the specificity of treatment of cancer cells while leaving healthy cells intact through the use of novel nanoparticles. This review discusses the use of nanoparticles such as quantum dots, nanoshells, nanocrystals, nanocells, and dendrimers for the detection and treatment of cancer. Future directions and perspectives of this cutting-edge technology are also discussed.

## 1. Introduction

**Figure 1.**
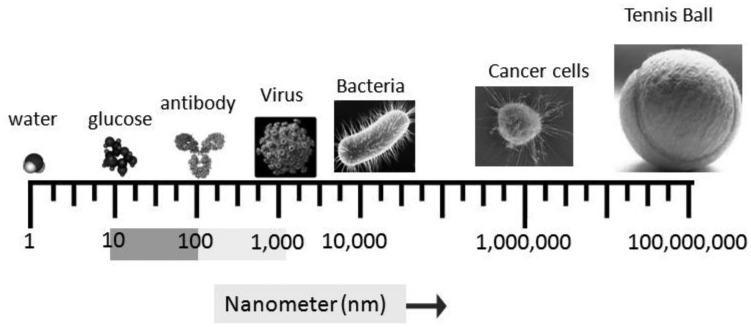
Relative sizes of different matters. “Nano” is from the Greek word for “dwarf” and means 10^−9^ meters or 1 nanometer (nm). The National Nanotechnology Initiative (NNI) defines nanotechnology at dimensions of roughly 1 to 100 nm (shaded scale region). Adapted from the National Cancer Institute (http://nano.cancer.gov/learn/understanding).

Nanotechnology is a broad word that comprises an assortment of subdisciplines in biology, biotechnology, engineering, chemistry, and physics. Categorically, nanotechnology includes all particles that are on the order of 1 billionth of a meter. The National Nanotechnology Initiative (NNI) defines nanotechnology at dimensions of roughly 1 to 100 nanometers (nm) [1], but many in the scientific community advocate that in terms of size, nanoparticles extend up to 1000 nm. More importantly, because of their nano-size, nanoparticles have unique physical and chemical properties that give them advantages as drug delivery carriers, or ‘nano-carriers’, and diagnosis probes. Additionally, at this size range, nanoparticles have a maximum surface:volume ratio, which is ideal for surface functionalization as well as incorporation of a therapeutics load. Furthermore, due to their nano-size and tunable surface properties (enabling the synthesis of aqueous, injectable solutions and the development of passive or active targeted systems), nanoparticles potentially have better access to tumor sites as compared to conventional drug delivery carriers (Figure 1).

**Figure 2.**
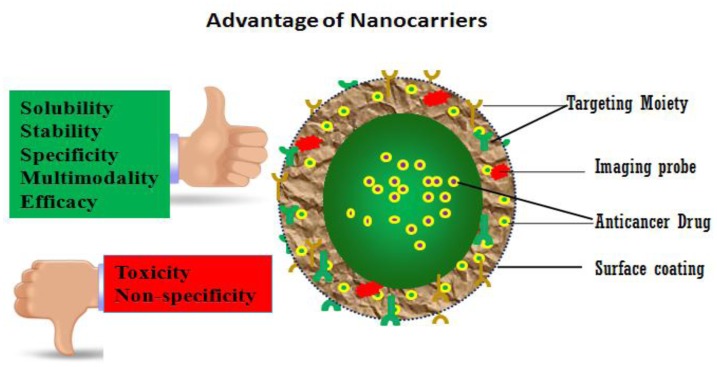
Nanoparticles as nano-carriers can increase solubility, stability, specificity, multimodality, and efficacy, while reducing toxic side effects and improving upon the non-specificity of conventionally delivered cancer treatments.

The application of nanotechnology is rapidly progressing, and has tremendous potential to make a revolutionary impact in healthcare, with profound effects on current treatment paradigms for various disease states. Thus, this unique technology might be a ray of hope in treating a complicated disease like cancer, a disease that accounted for up to 7,021,000 deaths in 2007 worldwide, and is the second leading global killer (12.5% of deaths) [2]. The World Health Organization predicted that cancer would be the utmost cause of death worldwide due to any single disease by the year 2010 [3]. Although scientists have made a relentless effort over the past few decades to contain cancer, current cancer treatment regimens consist of doses of compounds that are non-specific and highly toxic. Also, the inability of conventional diagnosis tools to detect cancer in an early and potentially curable stage further hinders effective treatment options, and thus by the time cancer is detected it may be too late to prevent metastasis to other organs in the body. Due to their unique physical and chemical properties, different nano-carriers have come forward as feasible solutions for many of the drawbacks associated with existing cancer treatments (Figure 2). Two key problems presently preventing effective cancer cures are: a) early detection of cancer before it metastasizes; and b) specific treatment of malignant cancer cells without affecting surrounding, normal tissues vis-à-vis avoiding unnecessary toxicity due to the inherent solubility problems associated with toxic organic solvents needed to solubilize the drug. The nanoparticles that are undergoing extensive study to determine the role that they might play in cancer detection and treatment include quantum dots, nanoshells, nanocrystals, nanocells, and dendrimers.

## 2. Nanotechnology and Cancer Detection

One of the most important factors in effective cancer treatment is the detection of cancerous tumor cells in an early and perhaps curable stage. Thus, the detection time frame has an enormous effect on a patient's prognosis. Nanotechnology brings new hope to the arena of cancer detection research, owing to nanoparticles' unique physical and chemical properties, giving them the potential to be used as a synthetic scaffold for imaging probes in the detection and monitoring of cancer. Nanoparticles' surface properties are tunable, meaning injectable solutions of them can be made without using toxic organic solvents to attach water-insoluble anticancer agents. This, along with nanoparticles' ability to do passive or active tumor targeting, makes them an excellent platform to use for diagnostic imaging and treatment. Thus, nanotechnology-based imaging modalities have made a significant entry into cancer research with their potential of highly sensitive probes for cancer detection [4].

## 3. Quantum Dots

Over the past few decades quantum dots (QDs) have been an area of intense research due to their unique physical properties that can be exploited for cancerous tumor detection. QDs usually consist of an inorganic transition metal core/shell system, and the majority of QDs are made up of cadmium selenide (CdSe), cadmium telluride (CdTe), indium phosphide (InP), and indium arsenide (InAs) as core elements inside a shell, usually zinc sulfide (ZnS). The major reasons that these inorganic-organic composite nanoparticles are extremely efficient agents for cancer detection *in vivo* are their small size, which gives them unhindered access to the systemic circulation, and at the same time their ability to conjugate targeting molecules that direct specific accumulation in neoplastic sites [5-7]. Additionally, similar to other nanoparticles, QDs have sufficient surface area to attach therapeutic agents and tumor-specific moieties for simultaneous drug delivery and *in vivo* imaging and tissue engineering [8]. Depending on size and the core/shell system, QDs have the ability to emit light across the visible and infrared wavelength spectrum, and thus one can choose a suitable color of light emission. The main advantage of the QDs is that with a single light source, the variously-sized QDs can be excited while preserving the narrow emission of each individual particle/wavelength [9]. Additionally, QDs have the ability to incorporate different markers simultaneously (multiplexing), enabling numerous targets to be imaged in a single experiment [10].

Initially, one of the major problems associated with QDs was their instability and water dispersability, but this was resolved by using various surface coatings, which not only increased the stability of the QDs, but also allowed incorporation of desirable tumor-targeting ligands for possible tumor detection. Such ligands include antibodies, peptides, and small-molecule drugs/inhibitors [11-13]. The use of QDs for *in vivo* cancer targeting and imaging in live mice was first reported by Gao *et al.* [6]. They showed the feasibility of *in vivo* imaging by subcutaneous injection of prostate cancer cells labeled with QDs. They also demonstrated the use of systemic injection of multifunctional QD probes that enable multicolor fluorescence imaging of cancer cells with high sensitivity [6]. Bagalkot and colleagues showed that QDs can be used for both imaging and therapy; QD-apatamer (Apt)-doxorubicin (Dox) conjugate was used for targeted cancer therapy and imaging of prostate cancer cells that express prostate-specific membrane antigen (PSMA) protein [14]. Significantly, these multifunctional QDs facilitated the targeted delivery and monitoring of doxorubicin release into tumor cells through activation of QDs as well as simultaneous imaging of the tumor tissue.

**Figure 3.**
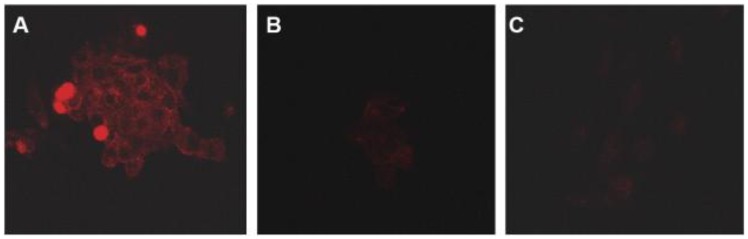
Confocal microscopy images showing: (**A**) uptake of QDs conjugated to an anti-PSMA antibody by LNCaP cells (a PSMA-positive cell line); (**B**) uptake of void QDs (without PSMA conjugation) by LNCaP cells; (**C**) uptake of anti-PMSA-conjugated QDs in PC-3 cells (a PSMA-negative cell line). For each condition, QDs were incubated with cells for 4 hours [15].

In an attempt to exploit the targeting ability of QDs, we recently synthesized anti-PSMA antibody-conjugated pegylated QDs (PSMA-QDs) [15]. Strepatvidin-biotin chemistry was used to attach the anti-PSMA antibody, where the antibody was first biotinylated using standard N-hydroxy succinimide conjugation chemistry, and then conjugated to amino-functionalized QDs. The uptake of PSMA-QDs with LNCaP prostate cancer cells (a PSMA-positive cell line) was more prominent compared to un-conjugated QDs. Also, in this *in vitro* study there was no detectable uptake of PSMA-QDs by PC-3 cells (a PSMA-negative cell line) (Figure 3).

**Figure 4.**
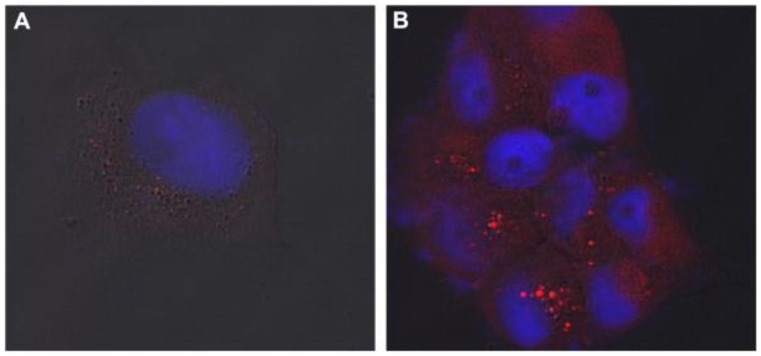
Confocal microscopy images showing uptake of Tetrac-PEG-QDs by Panc1 cells: (**A**) Cells were left untreated prior to incubation with QDs; (**B**) cells were pre-treated with T_4_ (thyroxin, a thyroid hormone) for 2 hours prior to the addition of QDs [16].

We also demonstrated the feasibility of the chemical conjugation of Tetrac (a thyroid hormone), known to have anticancer activity, to pegylated QDs (Tetrac-PEG-QDs) [16]. The chemical conjugation of Tetrac resulted in no loss of fluorescence to the QDs, and Tetrac-PEG-QDs were efficiently taken up by Panc1 pancreatic cancer cells (Figure 4). Nanoformulated Tetrac retained its anti-proliferative activity while conjugated to the QDs. These findings illustrate remarkable prospects of QDs for site-specific drug delivery and detection of various cancer cells.

A recent advancement in QDs technology is the use of QDs for near infrared (NIR) imaging (700–1000 nm wavelength range) as an imaging probe [17,18]. The main advantage of NIR QDs over its counterpart, visible QDs, is that it increases the depth of tissue penetration, allowing for more accurate and sensitive detection of photons *in vivo*. Additionally, NIR QDs evade the problem of auto-fluorescence associated with optical imaging because of the naturally-occurring compounds present in animal tissue. The use of NIR QDs for *in vivo* imaging was demonstrated for lymphatic mapping in animal models [19], and for biological imaging, using InAs/ZnCdS as a core/shell. NIR QDs coated with PEG allowed imaging of tumor vasculature as deep as 200 um, contrary to the visible QDs-generated images with very poor vascular contrast [20].

In summary, owing to their unique properties such as photostability, size- and composition-tunable emission properties (from visible to infrared wavelengths), and their ability to deliver multiple diagnostic or targeting agents, QDs have emerged as a promising nanotechnology for cancer detection. Furthermore, utilization of NIR QDs can potentially not only maximize the depth of tissue penetration compared to conventional imaging, but also can enhance the accuracy and photon detection sensitivity in an *in vivo* systems.

## 4. Iron Oxide Nanocrystals

Though there have been tremendous efforts to determine a suitable imaging tool for cancer detection, until now only magnetic resonance imaging (MRI) has been used. It is one of the most frequently-used, non-invasive imaging tools for disease diagnosis and monitoring, including cancer. However, a major problem associated with MRI is its low sensitivity. Utilization of nanotechnology to improve the sensitivity and efficacy of MRI for cancer detection and imaging is an area that researchers have focused on in the last several decades [21-23].

Magnetic nanoparticles used in biomedical applications mainly have an inorganic nanoparticle core and in most cases are coated by a suitable coating material [24-26]. Suitable coatings not only increase the stability and solubility of the nanoformulation but also can be used to incorporate a targeting moiety to increase the imaging sensitivity and to do real-time monitoring. Enhanced proton relaxation is one of the most added-value properties that make magnetic nanoparticles one of the best contrast agents for biomedical applications of MRI [27]. The most widely used nanoparticles of this kind are the super-magnetic iron oxide (SPIO) nanoparticles, which have been used under various trade names. SPION has been used as a bowel contrast agent (Lumerin, Gastromark) and for spleen/liver imaging (Endorem, Feridex) [22,28]. Combidex®, an ultra-small super-magnetic iron oxide (USPIO), represents one of the major successes in this class of nanoparticles. Combidex® is in late-stage clinical trials for the detection of lymph node metastases [29]. Imaging liver tumors is a specialty use of SPIO nanoparticles [30]. It was observed that Kupffer cells (hepatic microphages located in the hepatic parenchyma) can efficiently uptake these kinds of magnetic nanoparticles. Macrophage-specific uptake of SPIOs increases the contrast between healthy and diseased tissue because most liver tumors are devoid of it. Negative enhancement effects of SPIO nanoparticles on T2/T2*-weighted MRI sequences allowed increased lesion conspicuousness and increased lesion detection as compared to non-enhanced imaging. It is well documented that with the help of this technique, liver tumors or metastases as small as 2–3 mm can be detected. The first SPIO nanoparticles that were used in Europe for the detection of focal lesions in liver were Ferumoxides, 120-180 nm nanoparticles consisting of SPIO incorporated into T10-dextran. Since then, a variety of iron oxide-based nanoparticles of different sizes and with different coatings have been applied; some of them are already on the market [31]. Preliminary toxicity studies of these magnetic nanoparticles have proven that these nanoparticles are relatively safe for clinical use [32-34]. A list of magnetic nanoparticles in clinical trials and already on the market is presented in Table 1.

## 5. Nanotechnology and Cancer Treatment

The cure for cancer remains as an elusive goal. Though there have been countless drugs coming to the market with the promise of eliminating this lethal disease, most of these drugs have proved to be too toxic or simply not as effective at extending life expectancy as originally projected. Most of these drugs have serious side effects, even resulting in death to the patient, mainly because of their non-specificity, and thereby seriously affecting normal cells along with the tumor cells. One of the major strengths of a nanomedicine approach is the ability to alter the pharmacokinetics and biodistribution of the drug. The idea behind targeted delivery that is now being elucidated is that chemotherapy drugs can be directed to cancer cells by exploiting the same properties of cancer cells that made their detection and targeting possible.

The use of most chemotherapeutic agents is limited by their inherent problems such as poor solubility and low bioavailability, and the toxic solvents used to formulate them [35]. Nanotechnology might have a deep impact in solving many of the problems associated with conventional anticancer drugs because nano-formulated drugs can be made as relatively safe, injectable formulations. Doxil and Abraxane are the two major nano-formulated drugs currently available on the market and already they have made an impact in cancer treatment worldwide. Doxil, which is doxorubicin formulated in nano-liposome [36,37], has shown significant improvements over its counterpart, free doxorubicin. Abraxane® (Abraxis), with a size around 100 nm, is an albumin-bound nanoparticle formulation of paclitaxel [38-40] and is widely used for the treatment of metastatic breast cancer. The major advantage of Abraxane® is that it evades the hypersensitivity reaction associated with Cremophor EL, the solvent used in conventional paclitaxel therapy. Thus, Abraxane, clearly demonstrates the ability to convert insoluble or poorly soluble drugs, avoiding the need for toxic organic solvents. A list of the nanoformulations currently available on the market is in Table 2 [41].

Nanotechnology not only has the potential to conjugate the required targeting moiety, but also has the ability to carry the moiety for site-specific delivery without compromising its activity. Various polymeric materials are often used to synthesize nanoparticles loaded with conventional chemotherapy drugs such as docetaxel or doxorubicin, and then coated with polyethylene glycol to evade the patient's immune system. Additionally, nanoparticles can be conjugated with a targeted moiety such as an aptamer bioconjugate that binds, for example, to prostate-specific membrane antigens present on prostate cancer cells [42]. This type of active-targeted delivery to the tumor by using a tumor-specific moiety can be achieved by exploiting various natural interactions like lectin-carbohydrate, ligand-receptor, and antibody-antigen interactions within the tumor cell [43], resulting in preferential accumulation within the cancer-bearing organ or cancerous tumor cells. Active targeting has the potential to change current cancer treatment scenarios.

Some nanoparticles have the ability to accumulate in tumor vasculature, known as enhanced permeability and retention (EPR) [44-46], thus increasing accumulation of the payload to the tumor site. Passive targeting in this case takes advantage of the rapid vasculariztion of hyper-permeable cells. This results in leaky, defective vessels and impaired lymphatic drainage. Nanoparticles sized at 10 to 100 nm have the ability to accumulate within tumors because of their ineffective lymphatic drainage. Thus, consideration of the size and surface properties of nanoparticles is vital, particularly for passive targeting. Particles must be less than 100 nm to avoid uptake by the reticulo-endothelial system and their surface should be hydrophilic to avoid rapid clearance by macrophages [47]. Furthermore, both active and passive targeting can be exploited simultaneously to obtain maximum efficacy.

## 6. Gold Nanoshells

Gold nanoshells are useful in detecting tumors and metastasis in many solid tumors. The main advantage of the gold is its potential for cancer detection and treatment of cancers using near-infrared light. In a study where silica/gold nanoshells were used to treat breast cancer *in vivo* [48], the nanoshells were injected into the tumor site and irradiated with 820 nm, 4 W/cm^2^ light pulses. The tumor site increased in temperature when irradiated with light, and thus this system had the ability to destroy the tumor cells without causing any harm to the surrounding, normal cells. In another step forward, gold nanoshells were conjugated with ligands for specific accumulation in oral squamous carcinoma cell lines (HSC 313 and HOC 3 Clone 8) [49]. Furthermore, these kinds of nanoshells have been used for targeted delivery and therapy of many cancers, including breast and prostate cancers [48-50].

## 7. PLGA Nanoparticles/Nanocells

One of the most extensively used nanoparticles for cancer treatment is the poly (lactide-co-glycolide) (PLGA)-based nanoparticle. Proven biodegradability and a safe history have made PLGA nanoparticles a first choice for many researchers. Fonseca *et al.* reported encapsulation of paclitaxel in PLGA nanoparticles synthesized by interfacial deposition [51], and found an initial fast release profile during the first 24 hours of administration and later, a slower, continuous-release profile. Increased cytotoxicity of the nanoformulation was observed when it was compared to commercial formulations of free paclitaxel in an *in vitro* cell viability test in the NCI-H69 SCLC human small cell lung cancer cell line.

Synthesis of an oral formulation of paclitaxel using PLGA/montmorillonite (PLGA/MMT) nanoparticles has also been reported [51,52]. Cellular uptake of the fluorescently-labeled (coumarin-6-) PLGA/MMT nanoparticles had enhanced uptake efficiency for PLGA/MMT when compared with the PLGA nanoparticles by 57% to 177% for Caco-2 cells, and 11 to 55% for HT-29 cells, depending on the amount of MMT. The increased uptake was explained by the hypothesis that this formulation has a longer residence time in the gastrointestinal tract and potentially an increase in the oral absorption of paclitaxel. Additionally, PLGA/MMT nanoparticles can be used for targeted delivery to treat breast cancer [53]. The anti-human epidermal growth factor receptor-2 (HER2) antibody trastuzumab was conjugated to PLGA/MMT nanoparticles incorporating paclitaxel for targeted delivery. Using confocal microscopy, it was observed that there was a significant uptake of anti-HER2-conjugated PLGA/MMT nanoparticles compared to un-conjugated nanoparticles in both Caco-2 colon adenocarcinoma cells and SK-BR-3 breast cancer cells. The cytotoxicity of the antibody-conjugated nanoparticles encapsulating paclitaxel was 13-fold higher in SK-BR-3 cells when compared to free paclitaxel/un-conjugated PLGA/MMT nanoparticles [53]. Vitamin E-TPGS-emulsified PLGA nanoparticles encapsulating paclitaxel were used to study its efficacy in HT-29 cells and they also exhibited an initial ‘burst’ of release, followed by a sustained release mechanism *in vivo* [54]. Vitamin E-TPGS-emulsified PLGA nanoparticles encapsulating paclitaxel showed a better cytotoxic effect in HT-29 cells than commercially available paclitaxel. A 3-fold increase in the concentration of paclitaxel was found in *in vivo* pharmacokinetic measurements when compared to non-encapsulated drug.

The use of polyethylene glycol is one of the most common practices for passive targeting to the tumor site. A biocompatible and biodegradable nanoparticulate system using long circulating PLGA-monomethoxy-poly (polyethylene glycol) (PLGA-mPEG) nanoparticles has been synthesized [55]. The main advantage of this nanoparticulate system, encapsulating cisplatin, is its potential for passive cancer targeting. Cisplatin-doped PLGA-mPEG nanoparticles showed an initial rapid release of drug, followed by a relatively slow release phase *in vitro* at a pH of 7.4, as shown by most PLGA-based nanoparticles. However, it was observed that the release kinetics depend on the ratio of PLGA to mPEG. The amount of released cisplatin in the initial burst increases with the increase of the amount of mPEG in the nanoformulation. Intravenous administration in BALB/c mice of this nanoformulation incorporating cisplatin resulted in an increased cisplatin residence time in the systemic circulation [55]. The comparative cytotoxic effects of cisplatin were demonstrated when encapsulated in PLGA-mPEG nanoparticles on human prostate cancer LNCaP vs. free cisplatin [56].

Chemical conjugation of a chemotherapeutic drug to a nanoparticle carrier might be another solution for effective delivery of a drug. When doxorubicin was conjugated to PLGA nanoparticles in HepG2 human liver carcinoma cells, the nanoformulation slightly decreased the toxicity of doxorucin to HepG2 human liver carcinoma cells *in vitro*. However, the *in vivo* antitumor activity of the nanoparticles was similar to free doxorubicin [57,58]. PLGA nanoparticles incorporating doxorubicin coated with polysorbate 80 drastically increased the accumulation of doxorubicin in brain tissue [59]. The major disadvantage of these nanoparticles was found to be their acute renal toxicity [60].

## 8. Dendrimers

Dendrimers are a unique group of nanoparticles that are highly suitable for effective delivery of drugs, particularly for cancer treatment. Dendrimers can be synthesized by controlled, repeated polymerization reactions to engineer a desired shape and size. The main advantage of dendrimers is their exclusive branching point that is available for conjugation to multiple entities [61], including targeting proteins, treatment moieties, and even apoptosis factor ligands. Chemotherapy drugs, when incorporated into the core of the dendrimer, do not affect healthy cells [62]. The dendrimer can be engineered so that when it gets into the target tumor cell, it can change its conformation, allowing the incorporated moiety to be released to the tumor site, efficiently suppressing tumor growth. The size, tenability, and multifunctional capability to enhance multiple drug interactions to deliver a chemotherapeutic agent to the specific tumor site make dendrimers an excellent nano-carrier for tumor targeting and therapy [63,64].

Along with active targeting, another aspect of dendrimer-mediated delivery is achieved by passive targeting, mainly through pegylation on the dendrimer's surface. Folate-mediated site-specific delivery of nanoparticles [65] is one of the most commonly used methods because many cancer cells over-express folate receptor. This concept can be extended to dendrimers by surface conjugation. Folate-modified dendrimers target these cells via ligand-receptor recognition. A folic acid-incorporated dendrimer, covalently conjugated with methotrexate, specifically kills receptor-expressing cells after intracellular delivery of the drug through receptor-mediated endocyctosis [66]. Quintana *et al.* synthesized an ethylenediamine core PAMAM dendrimer of generation 5 that was covalently attached to folic acid, fluorescein, and methotrexate. This complex provided targeting, imaging, and intracellular drug delivery capabilities with 100-fold decreased cytotoxicity over free methotrexate [67].

## 9. Future Perspective

Nanotechnology is considered one of the greatest man-made engineering marvels in minuscule scale. The technology has grown exponentially in recent years, and it arguably has had the most impact on contemporary science and society since technologies of the Industrial Revolution. Demand for this cutting-edge technology in biomedical fields is growing by more than 17% annually, and is expected to reach approximately $53 billion by 2011 [68]. One prospective report predicted that in the near future half of pharmaceutical industry products will have some connection with nanotechnology [69].

Nanotechnology has already made an impact on cancer detection and therapy. The rapid intrusion of this cutting-edge technology in the current pharmaceutical industry is manifested by Abraxane, a nanomedicine approach to treat metastasis breast cancer. These aluminum-bound paclitaxel nanoparticles also have treatment potential for other cancers with or without the co-presence of other anticancer drugs. Many nanomaterials like SPIO and USPIO nanoparticles are extensively used under various trademarks for imaging of various types of cancers. On the website ClinicalTrials.gov, a registry of federally and privately supported clinical trials conducted in the US and around the world, it is revealed that over 70 nanomedicine approaches are currently in clinical trials for cancer treatment and imaging [70].

Though many of the technologies involving nanoparticles for cancer detection and treatment are mainly in preclinical stages, there is tremendous potential for nanotechnology to enable desperately-needed cancer detection in its early stages. Nano-carriers loaded with a chemotherapeutic payload targeting the tumor site can not only eliminate adverse side effects, but may also pave the way for bringing a more effective, specific, and personalized medicine for eradicating cancer and many other complex diseases. Thus, n nanotechnolgy has multifunctional proficiency and enormous potential to detect, treat, and monitor in real time. Nanotechnology applications in cancer detection and treatment have the potential to replace highly invasive conventional cancer detection and treatment, which often includes biopsies, irradiation, and painful therapies; they can become part of a painful past.

## Figures and Tables

**Table 1.** Magnetic nanoparticles in clinical trials or currently available on the market.

**Product**	**Company/Developer**	**Coating Agent**	**Application**	**Targeting Moiety**	**Use**
Feridex/ Endorem	AMAG Pharma, Inc.	Dextran	Liver tumors	None	Imaging
Ferumoxytol	AMAG Pharma, Inc.	Polysorbito carboxy methyl ether	CNS tumors	None	Imaging
Resovist®	Bayer Schering Pharma AG	Carboxydextran	Liver metastasis; colon cancer	None	Imaging
SPION	Sun, Ranganathan, Feng 2008	PEG/Dextran	Breast cancer	Folic Acid	Imaging
SPION	Kohler *et al.*, 2005	3-(aminopropyl) trimethoxysilane	Brain tumors	Methotrexate	Imaging and treatment
SPION	Sun, Lee, Zhang, 2008	PEG	Brain tumors	Chlorotoxin	Imaging and treatment
SPION	Wang *et al.*, 2008	PEG	Prostate cancer	A10 RNA aptamer	Imaging and treatment
SPION	Leuschner *et al.*, 2006	Chorionic gonadotropin	Breast cancer	LHRH	Imaging
SPION	Kikumori *et al.*, 2009	Liposome	Breast cancer	Anti-HER2 antibody	Imaging
SPION	Chen *et al.*, 2009	Dextran	Breast cancer	Herceptin	Imaging
USPION	Jiang *et al.*, 2009	3-(aminopropyl) trimethoxysilane	Lung cancer	RGD	Imaging

CNS, central nervous system; PEG, poly(ethylene glycol); LHRH, luteinizing hormone releasing hormone; RGD, arginine-glycine-aspartic acid.

**Table 2.** Nanoparticle formulations currently available on the market.

**Product**	**Company**	**Drug**	**Formulation/ROA**	**Application**	**Status**
Abraxane	Abrasix Bioscience, AstraZeneca	Paclitaxel	Albumin-bound nanoparticles/iv	Metastatic breast cancer	Marketed
Caelyx	Schering-Plough	Doxorubicin	Pegylated liposome/im	Metastatic breast and ovarian cancer; Kaposi sarcoma	Marketed
Myocet	Zeneus Pharma Ltd	Doxorubicin	Liposome/iv	Metastatic breast cancer	Marketed
Doxil	Sequus Pharmaceutical	Doxorubicin	Liposome/iv	Kaposi sarcoma	Marketed
L-Annamycin	Callisto Pharmaceuticals	Annamycin	Liposome/iv	Children and young adults with refractory or relapsed ALL or AML	Phase I/II
Genexol-PM	Samyang Pharmaceuticals	Paclitaxel	Methoxy PEG-PLA/iv	Breast and lung cancer	Phase II
CALAA-01	Calando Pharmaceuticals	Anti-R2 SiRNA	Cyclodextrin-containing polymer (CAL 101) and targeting agent (AD-PEG-Tf)/iv	Solid tumors that are refractory to standard-of-care	Phase I
Rexin-G	Epeius Biotechnologies	Dominant negative cyclin G1 construct	Pathotropic nanoparticles/iv	Recurrent or metastatic breast cancer	Phase I/II
*BikDD* Nanoparticle	MD Anderson Cancer Center/NCI	Pro-apoptotic Bik gene (*BikDD*)	Liposome/iv	Pancreatic Cancer	Phase I
Docetaxel-PNP	Samyang	Docetaxel	Polymeric nanoparticles/iv	Advanced solid malignancies	Phase I

ROA, route of administration; iv, intravenous; im, intramuscular; ALL, acute lymphocytic leukemia; AML, acute myelogenous leukemia; PEG-PLA, poly(ethylene glycol)-poly(lactide); Tf, human transferring protein; HCC, hepatocellular carcinoma
